# Research advances in replication-deficient viral vector vaccines

**DOI:** 10.3389/fvets.2025.1535328

**Published:** 2025-03-03

**Authors:** Junna Wang, Jin Cui, Guoxin Li, Lingxue Yu

**Affiliations:** ^1^Shanghai Veterinary Research Institute, Chinese Academy of Agricultural Sciences, Shanghai, China; ^2^Shanghai Key Laboratory of Veterinary Biotechnology, Shanghai, China; ^3^College of Veterinary Medicine, Northeast Agricultural University, Harbin, Heilongjiang, China

**Keywords:** replication-deficient virus, replication-deficient vaccine, single-cycle replication, safety, vector, vaccine

## Abstract

In recent years, replication-deficient viral vector vaccines have attracted much attention in the field of vaccine research and development due to their high safety and immunogenicity. These vaccines use genetic modifications to engineer viral vectors that make them unable to replicate but effective in expressing recombinant proteins and induce immune responses. Currently, replication-deficient adenovirus vectors and poxvirus vectors are widely used in vaccine R&D for a variety of infectious diseases in humans and animals, including AIDS, hepatitis B, pseudorabies, avian influenza, infectious bronchitis in poultry, and foot-and-mouth disease. Replication-deficient viral vaccines have been shown to effectively induce neutralizing antibodies and cellular immune responses, thereby providing effective immune protection. Future development of genetic engineering technology and continuous in-depth research on viral vectors should lead to replication-deficient viral vector platforms that have an essential role in preventing and controlling existing and emerging infectious diseases.

## 1 Viral vector vaccines

Viral vector vaccines utilize modified viruses, including recombinant attenuated and non-host viruses to deliver antigens, thereby eliciting an immune response (1). These vaccines have demonstrated efficacy across various applications, with notable examples including adenovirus [Adv; (2)], modified vaccinia virus (pox) (3), and turkey herpesvirus [HVT; (4, 5)]. However, traditional live attenuated viral vector vaccines face challenges mainly safety concerns, such as potential reversion to virulence, and uncontrolled shedding into the environment (6, 7). Replication-defective vectors address these issues by enhancing safety, making them an ideal choice for the next generation of vaccines.

## 2 Replication-deficient viruses

Replication-deficient viruses are genetically modified viruses lacking functions essential for the replication of one or more viral genomes or virion synthesis and assembly in which the critical replication gene regions in the genomes are dysfunctional (8). Such viruses cannot complete the replication process independently and must rely on exogenous proteins provided by the host cell for assembly into mature viral particles. Once infecting host cells, they can only undergo one round of infection without continuing to replicate themselves, and this dependence explains why replication-deficient viruses exhibit a high level of biosecurity. In normal cells, one or more steps of viral replication are blocked, resulting in viral gene expression within infected cells without producing progeny virus (9). The tight blockade of these mutant viruses in replication makes their high level of safety one of the major advantages of using replication-deficient mutant viruses as vaccine vectors.

Current research on replication-deficient viruses also includes the study of single-cycle mutant viruses. By contrast, a single-cycle infectious virus is somewhat similar to a viral vaccine but is technically different. It is defective in the virion proteins that function after virus assembly and multiplies in complementary cells that express the deletion gene product. First infection, the replication cycle proceeds normally and produces progeny virions. However, since these virions are non-infectious, the infection cannot spread to the second round of cells, but the released non-infectious virions may provide inert antigens that can spread beyond the infected cells, thereby potentially leading to incomplete blocking of viral transmission (10).

## 3 Replication-deficient virus vectors and vaccines

### 3.1 Adenovirus

Replication-deficient recombinant adenoviruses are currently the most widely investigated and applied replication-deficient vaccine vectors. Adenoviruses are non-enveloped double-stranded DNA viruses with a genome length of ~36 kb in which the first generation adenoviral vectors become replication-deficient by deleting the E1 and E3 genomic proteins necessary for viral replication and gene expression while inserting transgene expression sites, resulting in high immunogenicity (11, 12). Second generation adenoviral vectors further delete the E2 and E4 genomic regions, increasing their carrying capacity while maintaining high immunogenicity (13, 14), and third generation adenoviral vectors delete the entire adenoviral genome while retaining only the essential components to achieve high levels of gene expression but requiring helper adenoviruses for packaging (15). In this regard, the main drawback of adenoviral vectors is that the first generation adenoviral vectors, which lack the E1 protein and are the most widely used, can only insert up to 9 kb of exogenous gene sequences (16). Despite the ability of second and third generation vectors to accommodate larger exogenous gene sequences because more viral genes are deleted, the viral titer and expression levels of the exogenous genes are significantly lower than those of the first generation adenoviral vectors [(17, 18); Table 1].

**Table 1:** The replication-deficient adenoviral vectors.

**Generation**	**Characteristics, pros, and cons**	**Maximum transgene capacity**	**Vaccine applications (references)**
First generation	Deletion of E1 and E3 genes, retention of E2 and E4 genes; Pros: high immunogenicity, high viral titer, and high transgene expression levels; Cons: limited insertion of exogenous genes	9 kb	COVID-19 vaccine: ChAdOx1 (12)
Second generation	Further deletion of E2 and/or E4 genes based on the first generation; Pros: longer transgene expression duration; Cons: weaker immune response	14 kb	Human α1 antitrypsin (hAAT) -E2a-deleted adenoviral vector (14)
Third generation	Deletion of most or all adenoviral genes, retaining only ITR and packaging signals; Pros: Introduce extended exogenous genes; Cons: reduced cellular immune response	35 kb	Zika vaccine: Ad26 vector (38)

Replication-deficient recombinant adenovirus vaccines inhibit the progression of chronic Chagas's disease and reverse cardiac injury by reprogramming immune responses (19). A vaccine for blocking the transmission of malaria (AdPvs25) was developed by use of replication-deficient human adenovirus type 5 (rAd5) as a vector to express the surface protein (Pvs25) of the malignant *Plasmodium* parasite, and immunizing mice with this vaccine effectively induced antibodies against Pvs25. Compared with recombinant Pvs25 protein vaccine mixed with aluminum adjuvant, AdPvs25 had higher efficacy (20). Numerous studies have reported the application of Ad5 vector vaccines in human immunodeficiency virus type 1 (HIV-1), and Ad5 vaccines can induce potent and long-lasting CD8^+^ T-cell responses and specific cytotoxic T-cell responses (21). Additionally, other researchers found that a combination of DNA vaccine immunization followed by immunization with a replication-deficient adenoviral vector HIV-1 vaccine (Ad-Bal) induced higher humoral and cell-mediated immune responses (22).

The coronavirus disease 2019 (COVID-19) pandemic caused by the SARS-CoV-2 coronavirus in 2019 posed a significant challenge to healthcare systems worldwide, and investigators constructed a vaccine (Ad5-nCoV) that used the Ad5 adenoviral vector to express the S protein of SARS-CoV-2, assessed the vaccine's immunogenicity and protective efficacy in BALB/c mice and ferrets as animal models, and compared two different routes of administration: intramuscular injection and mucosal inoculation. The results indicated that a single dose of mucosal vaccination protects mice and ferrets from infection and inhibits virus replication in the upper respiratory tract. In comparison, intramuscular immunizations is insufficient to provide complete protection for the upper respiratory tract of mice against viral infections [(23); Table 2].

**Table 2:** A summary of examples of defective virus vaccines.

**Viral vector**	**Antigen/Test content**	**Vaccine status**	**References**
Adenovirus (rAd5)-human	Malaria Pvs25 protein	Research phase	(20)
	HIV-1 gag protein	Research phase	(21)
	SARS-CoV-2 S protein (Ad5-nCoV)	Research phase	(23)
	Ebola virus GP protein	Research phase	(25)
Gorilla adenovirus (GRAd)	SARS-CoV-2 S protein (GRAd-COV2)	Clinical trial	(32)
Chimpanzee adenovirus (ChAd)	Rabies virus G protein (ChAd155-RG)	Research phase	(33)
Flavivirus (rAds)	Measles virus, tick-borne encephalitis virus Rad-encoded non-structural proteins	Research phase	(41)
ChAd3-RSV; MVA-RSV	Respiratory syncytial virus (RSV) antigen	Research phase	(46)
Alphavirus	CMV glycoprotein B and pp65/IE1 fusion protein	Research phase	(52)
Herpes simplex replication-defective mutant viruses	Glycoprotein D	Research phase	(73)
Herpes simplex virus (HSV-2)	HSV-2 mutant	Research phase	(74)
Herpes simplex replication-defective mutant viruses	UL5/UL29-deleted HSV-2 mutant	Research phase	(75)
Replication-defective HSV-1 mutant	Replication-Defective HSV-1	Research phase	(76)
Adenovirus human	Feline coronavirus N protein (AD5-N)	Research phase	(58)
	Avian influenza HA gene	Research phase	(59)
	Infectious bronchitis virus S1 gene (rAd-S1)	Research phase	(60)
	Rabbit hemorrhagic disease virus VP60 protein (Ad-VP60)	Research phase	(61)
	Four potential protective antigens of *Francisella tularensis*	Research phase	(62)
Human adenovirus	Bovine herpesvirus gC/gD glycoproteins	Research phase	(63)
Recombinant bovine adenovirus vector	BHV-1 gD	Research phase	(64)
Human adenovirus	IHNV G protein and IPNV VP2	Research phase	(65)
	Classical swine fever virus (CSFV) E2 glycoprotein	Research phase	(66)
	Foot-and-mouth disease virus (FMDV) capsid-coding region	Research phase	(67)
	FMDV-Ad5-CI-A24-2B	Research phase	(68)
	Pseudorabies virus (PRV) antigen	Research phase	(69)
PRRSV ΔORF2/ΔORF4	Porcine reproductive and respiratory syndrome virus	Research phase	(79)
FCV ΔLC	Feline calicivirus VP1 protein	Research phase	(81)

Adenoviruses derived from non-human primates (NHP) share a similar genomic structure with human adenoviruses, albeit with some differences, such as sequence diversity in the major capsid proteins and E3 region (24). Compared with those of human adenoviruses, the immune responses induced by NHP adenoviruses may be more versatile and generate more memory T cells, which could help enhance the persistence of antitumor immunity. In this regard, NHP adenoviruses can serve as a valuable resource for developing novel oncolytic adenoviral vectors (24).

Researchers have developed vaccines based on human adenovirus type 5 (rAd5) that can provide protection against acute lethal Ebola virus (EBOV) challenges in macaques (25). However, such vaccines fail to protect macaques with preexisting Ad5 antibodies, indicating that prior infection with wild-type Ad5 may limit the vaccine's immunogenic efficacy in humans. Although adenoviruses from non-human primates (including chimpanzees and apes) may overcome this limitation and have shown promise for protection against the EBOV, the effect is limited to modified viruses in mice (25).

Adenoviruses have been isolated from chimpanzees, bonobos, gorillas, and macaques (26). Replication-deficient vector vaccine have been developed on the basis of the adenovirus backbone from gorillas and chimpanzees (27–31). One candidate vaccine, GRAd-COV2, is based on a replication-deficient gorilla adenoviral vector that stably expresses the spike protein of the SARS-CoV-2 virus. The results from postvaccination clinical trials showed that GRAd-COV2 was well-tolerated at all doses, with most participants in young and elderly groups producing sustained specific antibodies that neutralized variant strains. Additionally, they also produced potent T-cell responses targeting the spike protein, with mild and short-lasting side effects (32). A chimpanzee-derived replication-deficient adenovirus (ChAd) was utilized to express the glycoprotein (G protein) of the rabies virus, followed by the development of a novel rabies vaccine, ChAd155-RG, for serotype C, the administration method for which is simpler than existing methods. In addition, the vaccine exhibited a robust and enduring neutralizing antibody response in non-human primates, demonstrating superior protective efficacy (33).

The broad applicability of adenoviral vectors is further exemplified in the development of flavivirus vaccines (34, 35). Members of the Flavivirus family are responsible for various diseases in humans, including dengue fever, yellow fever, Japanese encephalitis, tick-borne encephalitis, Zika virus, and St. Louis encephalitis, etc. (36). Replication-deficient recombinant adenoviruses (rAds) have potential as flavivirus vaccines (37–40). For instance, animal model experiments using rAd vaccines for the measles virus and tick-borne encephalitis virus have induced good humoral and cellular immune responses, ultimately protecting animals from viral infections. rAds can express non-structural proteins of flaviviruses, effectively stimulating strong cellular immune responses chronic viral infections (41).

### 3.2 Poxvirus

Poxvirus is a large, enveloped double-stranded DNA virus with a genome size of 130–230 kb, and the prototype virus is the causative agent of smallpox (42). The Modified Vaccinia virus Ankara (MVA) strain, derived from an attenuated vaccinia virus strain, is a highly effective and safe replication-deficient viral vector platform (3). Previous studies on recombinant MVA vaccines have confirmed their protective efficacy, indicating the promising potential of this platform for vaccine development (43–45). Research has involved the vaccination of healthy adults with two different replication-deficient viral vector vaccines: the African green monkey adenovirus, ChAd3-RSV, and the Ankara vaccinia virus, MVA-RSV, both targeting respiratory syncytial virus (RSV; Table 2). Experimental data suggest that the MVA-RSV vaccine has immunogenicity and safety comparable to those of the ChAd3-RSV vaccine in healthy adults and can effectively induce humoral and cellular immune responses (46). The avian poxvirus is unable to produce progeny viruses when infecting mammalian cells (47).

### 3.3 Alphavirus

Alphavirus is a member of the Togaviridae family (48), characterized as an enveloped virus with a single-stranded positive-sense RNA genome. The non-structural genes are translated from the genomic RNA once it is released into the cytoplasm, forming replication complexes that drive the production of negative-sense antigenomic RNA. This process also involves the transcription of full-length positive-sense genomic RNA and shorter subgenomic RNA. As genome-wide RNAs are replicated, more templates are transcribed via subgenomic promoters, leading to the exponential amplification of subgenomic RNAs and rapid proliferation of subgenomic mRNAs (49). The use of alphavirus vaccine vectors is increasing because of their broad range of host cells, tropism for dendritic cells and monocytes, high levels of gene expression capability (50), and immunity to very few individuals, which makes them very promising candidates for replication-deficient vectors (51).

Investigators have used a replication-deficient, single-cycle, single-capsid alphavirus replicon vector system (52) to construct viral-like replicon particles expressing the extracellular domains of cytomegalovirus glycoprotein B and pp65/IE1 fusion proteins, which induced high-titer antibody responses and strong cellular immune responses in mice and rabbits while demonstrating satisfactory immunogenicity and safety [(53); Table 2].

## 4 Replication-deficient adenovirus vector vaccines in veterinary medicine

In recent years, various viral vaccines for animal diseases have been continuously developed to create safer and more effective novel vaccines (54). Replication-deficient adenovirus vaccines have demonstrated higher safety and more potent immunogenicity than traditional vaccines in clinical prevention and protection against many diseases, with several adenovirus vector vaccines from different species exhibiting superior immunological effects in immunization experiments conducted in mice and other animals (55). The main viral vectors include human adenovirus, chimpanzee adenovirus, and avian adenovirus, and there have been numerous applications in animal diseases beyond humans, such as swine influenza virus, porcine epidemic diarrhea (56), small ruminant viral diseases (57), and foot-and-mouth disease.

### 4.1 FCoV

Previous laboratory studies on feline infectious peritonitis (FCoV) found limited efficacy in the current vaccines against FCoV, primarily due to antibody-dependent enhancement in affected cats, and the nucleocapsid (N) protein of FCoV was constructed as a novel adenovirus vector (AD5-N) to develop a new vaccine to address this issue (Table 2). Compared with the control group, the AD5-N immunized animals group exhibited a strong cellular immune response and specifically induced high levels of IgG and SIgA antibodies, as well as showed evidently reduced viral loads in their feces and intestinal tissues after challenge assays. The experimental results indicated that the AD5-N vaccine provided effective protection for cats against FCoV infection (58).

### 4.2 AIV and IBV

Regarding avian diseases research, investigators have inserted the HA genes of different subtypes of avian influenza virus (AIV) into the HAdV5 vector, constructing replication-deficient adenoviruses that express various subtype genes (Table 2). In mouse models, polyvalent adenovirus vaccines can induce broad-spectrum immune responses and provide cross-protection against multiple subtypes of avian influenza virus (59). Aside from avian influenza viruses, the currently used infectious bronchitis virus (IBV) vaccines for poultry encounter issues, such as low cost-effectiveness and insufficient protective efficacy, particularly against nephropathogenic IBV (Table 2). In view of this, a recombinant adenovirus vaccine (rAd-S1) expressing the S1 gene of nephropathogenic IBV that effectively induces humoral and cellular immune responses in vaccinated chickens while producing antibodies and cytokines against IBV has been developed. Compared with the control group, chickens in the rAd-S1 group exhibited significantly relieved renal lesions and clinical symptoms after challenge assays, with evident decreased mortality (60).

### 4.3 RHDV and tularemia

Regarding infectious diseases in rabbits, some researchers have constructed an adenovirus vector vaccine (Ad-VP60) that efficiently expresses the VP60 protein of rabbit hemorrhagic disease virus (RHDV; Table 2). This Ad-VP60 vaccine exhibits stronger immunogenicity and induces high levels of anti-RHDV antibodies in immunized mice and rabbits. Additionally, rabbits immunized via the nasal route showed saliva containing IgA antibodies comparable to those found in sera from rabbits vaccinated with inactivated RHDV vaccines (61). Rabbit fever, also known as rabbit plague or Tularemia, is a naturally occurring infectious disease caused by *Francisella tularensis*, which is a highly contagious Gram-negative pathogen capable of infecting various mammals, including rodents, wild rabbits, domestic rabbits, and humans. Researchers have developed a subunit vaccine and an adenovirus vector vaccine expressing four potential protective antigens from *F. tularensis* (Table 2). In mouse experiments, the adenovirus vector vaccine was able to induce protective immunity after a single immunization and stimulated an immune response characterized by Th1 cell cytokines, which had significantly higher protective efficacy than the subunit vaccine (62).

### 4.4 BHV

Investigators have constructed a vaccine by inserting glycoprotein C (gC) or glycoprotein D (gD) of bovine herpesvirus type 1 (BHV-1) into the HAd5 vector (Table 2), followed by assessing its protective efficacy in rabbits and cattle through intranasal and intramuscular administration, with intranasal injection in rabbits showing higher BHV-1 neutralizing antibody levels than those in the intramuscular group and the intranasal injection in cattle showing higher levels than those in the commercial live vaccine group (63). Additionally, there are some reports of modifying bovine adenovirus to express BHV-1 gD, constructing replication-capable and -deficient recombinant bovine adenoviruses (Table 2). Animal experiments found that the replication-capable virus provided partial protection and reduced shedding without completely preventing BHV-1 infection. By contrast, the replication-deficient virus could stimulate an immune memory response without providing protection (64).

### 4.5 IHNV and IPNV

For aquatic animals, prevention of infectious hematopoietic necrosis virus (IHNV) and infectious pancreatic necrosis virus (IPNV) in rainbow trout is critically important. Investigators successfully coexpressed the G protein of IHNV and the VP2 protein of IPNV by inserting them into a recombinant adenovirus vector (Table 2). After immersion immunization of juvenile fish, the immune levels were substantially elevated, producing high levels of neutralizing antibodies against IHNV and IPNV. Challenge tests indicated that the recombinant adenovirus vaccine exhibited high protection rates against IHNV and IPNV, with relative survival rates of 81.25% and 78.95%, respectively (65).

### 4.6 CSFV

Immunogenic results from an adenovirus vaccine expressing the E2 glycoprotein of classical swine fever virus (CSFV) in pigs supported the construction of a recombinant adenovirus rAd-E2-InvC by coexpressing the C-terminal domain of the invasion protein (InvC) from *Yersinia enterocolitica* with the E2 glycoprotein (Table 2). Pigs immunized with rAd-E2-InvC did not show detectable virus or pathological changes upon necropsy after infection with CSFV, demonstrating complete resistance to virulent infection. By contrast, pigs in the rAd-E2 group exhibited clinical symptoms and viral nucleic acids, indicating that the coexpression group can enhance the efficacy of HAdV-based CSFV vaccines (66).

### 4.7 FMDV

Investigators also have inserted the capsid-coding region of foot-and-mouth disease virus (FMDV) into an adenovirus vector and added an Arg-Gly-Asp sequence to fibronectin, and the fiber-modified adenovirus vector vaccine was able to express FMDV capsid proteins more effectively *in vitro*, particularly in cells lacking the Coxackie and adenovirus receptor (Table 2). However, no significant differences were observed in protective efficacy between the 2 vaccine types in bovine *in vivo* experiments (67). Additionally, Poly ICLC was used as an adjuvant in combination with a replication-deficient human adenovirus vector FMD vaccine (Ad5-CI-A24-2B). After immunization and challenge, the animals showing no detectable FMDV-specific neutralizing antibodies were still protected because Poly ICLC enhanced the specific T-cell response. Given this, Poly ICLC can serve as an adjuvant for the Ad5-FMD vaccine, reducing the vaccine dosage, improving immunogenicity, and lowering production costs (68).

### 4.8 PRV

Other studies have also found that piglets immunized via intramuscular injection with a recombinant adenovirus (rAd) vaccine for pseudorabies virus (PRV) could effectively resist PRV infection (Table 2), regardless of whether or not the dam was immune. However, piglets vaccinated intranasally were poorly protected. Particularly, the intramuscular administration of the rAd vaccine effectively induced anti-PRV immune responses in newborn piglets, even in the presence of maternal antibodies (69).

## 5 Replication-deficient virus vaccine

### 5.1 HSV

Herpes simplex virus (HSV) is a common human disease pathogen that causes mucosal infections, spreads to sensory neurons, and establishes latent infections, which lead to recurrent diseases (70). In particular, HSV infections occur throughout the carrier's lifetime and manifest as various clinical syndromes, including herpes labialis, herpetic whitlow, eczema herpeticum, keratitis, encephalitis, and genital diseases (71). In individuals with compromised immune systems, such as patients with AIDS, HSV infections can lead to severe illnesses (72).

Herpes simplex virus type 2 (HSV-2), a sexually transmitted virus, is the most common cause of genital diseases worldwide. There is a current lack of effective treatment options, making vaccine development crucial. Various forms of replication-deficient mutant HSV-2 viruses have been tested as vaccine strains. In mouse models of HSV-2 infection, the protective effect of live vaccines was proportional to the vaccination dose. With increasing doses, viral replication in the reproductive tract, disease severity and mortality rates were remarkably reduced (73). Another group of investigators compared the protective effects of a replication-deficient vaccine and a subunit vaccine via different immunization routes, and the replication-deficient vaccine had significant advantages in protecting vaginal mucosa and dorsal root ganglia from infection. Additionally, intramuscular injection produced immunogenicity and protective effects higher than those of subcutaneous injection in genital HSV-2 infection models (74). Moreover, an assessment of the protective efficacy of replication-deficient vaccines lacking viral replication genes (UL5 and UL29) and subunit vaccines in guinea pigs and rabbits indicated good safety and tolerability (75). Subcutaneous immunization with replication-deficient herpes simplex virus type 1 (HSV-1) mutants has also been found to reduce viral infections in the cornea and latent infections in the trigeminal ganglia while inducing persistent immunity. They exhibited good safety and avoided the risks associated with replication-competent viruses (76).

### 5.2 PRRSV

Currently, PRRSV vaccines (77) have issues with safety (live attenuated vaccines may regain virulence) and efficacy [unsatisfactory efficacy of inactivated vaccines; (78)]. Since the deletion of ORF2 and ORF4 genes results in the virus being unable to replicate in MARC-145 cells and porcine alveolar macrophages, investigators successfully constructed two full-length infectious cDNA clones of gene-deleted viruses: ΔORF2-PRRSV and ΔORF4-PRRSV. At the same time, two complementary cell lines were constructed to stably express GP2 and GP4 proteins to rescue the viruses lacking these genes. Additionally, although pigs immunized with ΔORF2-PRRSV and ΔORF4-PRRSV showed reduced viral loads after challenge assays, there was no significant improvement in their clinical symptoms (79).

### 5.3 ASFV

Notably, African Swine Fever (ASF) is a highly contagious viral disease that causes substantial economic losses in the global pig industry. The ASF virus has multiple genotypes, and the high variability of its genes presents great challenges for vaccine protection. Despite the ability to induce immune responses and provide long-term protection against homologous ASFV infections, live attenuated ASFV vaccines fail to protect against heterologous strains, resulting in very limited protection. Additionally, attenuated strains developed by deleting virulence-related genes, such as ASFV-G-ΔI177L/ΔLVR and BA71ΔCD2, which can stably replicate in COS-1 cell lines and provide cross-protection are already known (80).

### 5.4 FCV

Reports have indicated the successful rescue of a virus containing the genome-wide feline calicivirus (FCV), named rBAC-FCV, with deletions made to the LC gene associated with FCV virulence, resulting in the infectious clone pBAC-FCV-ΔLC. Virus rescue using the feline kidney cell line F81-VP1, which expresses the FCV-VP1 protein, was conducted, followed by obtaining the LC gene-deleted strain rBAC-FCV-ΔLC. Additionally, cell experiments confirmed that deletion of the LC gene resulted in reduced viral virulence, and the virus lacking the LC gene could only replicate in cell lines that provide the VP1 protein, thereby significantly enhancing its safety. In conclusion, the successful rescue of this replication-deficient virus supports the use of alternative vaccine strains for developing novel FCV vaccines based on reverse genetics technology (81).

## 6 Application prospects of replication-deficient vaccines

Vaccines are one of the most critical tool-kit to prevent infectious diseases. There are various types of existing vaccines available commercially (82); traditional inactivated vaccines are considered safe, but often fail to elicit strong T-cell responses (83), whereas live attenuated vaccines can produce potent antibody and cellular immune responses, but carry potential risks of causing disease (84). By contrast, viral vector vaccines deliver genes from pathogens via viral vectors, to stimulate immune responses (85).

In recent years, replication-deficient viral vaccines have gained increasing attention. In the field of animal health. For example, there are currently no therapeutic drugs for feline infectious peritonitis, which makes the application of replication-deficient adenoviral vector vaccines that have demonstrated high levels of protection in animal experiments a promising research area. Moreover, aside from common replication-deficient adenoviral vector vaccines, single-cycle adenoviral vector vaccines induce higher antibody levels and more potent immune responses in animal experiments for EBOV (86). If these findings are confirmed, single-cycle adenoviral vector vaccines might have significant potential for infectious disease vaccines.

Currently, the commercially available PRRS (87) vaccines are mainly inactivated vaccines and live attenuated vaccines, neither of which achieves a balance between protective efficacy and safety (88). However, a significant portion of the strains in pigs are recombinant viruses derived from vaccine strains (89), and safety is the greatest advantage of developing replication-deficient vaccines (90) that can also stimulate humoral and cellular immunity in immunized animals, thereby compensating for the shortcomings of existing vaccines.

## 7 Concluding remarks and future perspectives

In conclusion, replication-deficient vaccines represent a transformative advancement within the rapidly evolving field of vaccinology. Through targeted genetic engineering, these platforms are being refined to achieve an optimal balance between immunogenicity and biosafety. As our understanding of pathogen biology and host immunity expands-complemented by breakthroughs in gene-editing technologies such as CRISPR and prime editing-replication-deficient vaccines are poised to assume a central role in mitigating both emerging and re-emerging infectious diseases.

Looking forward, the convergence of precision gene editing, structural virology, and computational immunology will propel the development and application of replication-deficient viral vectors and vaccines to address existing and emerging infectious disease.
